# *Loxhd1* Mutations Cause Mechanotransduction Defects in Cochlear Hair Cells

**DOI:** 10.1523/JNEUROSCI.0975-20.2021

**Published:** 2021-04-14

**Authors:** Alix Trouillet, Katharine K. Miller, Shefin Sam George, Pei Wang, Noor-E-Seher Ali, Anthony Ricci, Nicolas Grillet

**Affiliations:** ^1^Department of Otolaryngology-Head and Neck Surgery, School of Medicine, Stanford University, Stanford, California 94305; ^2^Department of Molecular and Cellular Physiology, School of Medicine, Stanford University, Stanford, California 94305

**Keywords:** hair cell, LOXHD1, mechanotransduction, nonsense-associated altered splicing, PLAT domain, stereocilia

## Abstract

Sound detection happens in the inner ear via the mechanical deflection of the hair bundle of cochlear hair cells. The hair bundle is an apical specialization consisting of actin-filled membrane protrusions (called stereocilia) connected by tip links (TLs) that transfer the deflection force to gate the mechanotransduction channels. Here, we identified the hearing loss-associated *Loxhd1/DFNB77* gene as being required for the mechanotransduction process. LOXHD1 consists of 15 polycystin lipoxygenase α-toxin (PLAT) repeats, which in other proteins can bind lipids and proteins. LOXHD1 was distributed along the length of the stereocilia. Two LOXHD1 mouse models with mutations in the 10th PLAT repeat exhibited mechanotransduction defects (in both sexes). While mechanotransduction currents in mutant inner hair cells (IHCs) were similar to wild-type levels in the first postnatal week, they were severely affected by postnatal day 11. The onset of the mechanotransduction phenotype was consistent with the temporal progression of postnatal LOXHD1 expression/localization in the hair bundle. The mechanotransduction defect observed in *Loxhd1*-mutant IHCs was not accompanied by a morphologic defect of the hair bundle or a reduction in TL number. Using immunolocalization, we found that two proteins of the upper and lower TL protein complexes (Harmonin and LHFPL5) were maintained in the mutants, suggesting that the mechanotransduction machinery was present but not activatable. This work identified a novel LOXHD1-dependent step in hair bundle development that is critical for mechanotransduction in mature hair cells as well as for normal hearing function in mice and humans.

**SIGNIFICANCE STATEMENT** Hair cells detect sound-induced forces via the hair bundle, which consists of membrane protrusions connected by tip links. The mechanotransduction machinery forms protein complexes at the tip-link ends. The current study showed that LOXHD1, a multirepeat protein responsible for hearing loss in humans and mice when mutated, was required for hair-cell mechanotransduction, but only after the first postnatal week. Using immunochemistry, we demonstrated that this defect was not caused by the mislocalization of the tip-link complex proteins Harmonin or LHFPL5, suggesting that the mechanotransduction protein complexes were maintained. This work identified a new step in hair bundle development, which is critical for both hair-cell mechanotransduction and hearing.

## Abstract (precis)

Sound detection happens in the inner ear via the mechanical deflection of the hair bundle of cochlear hair cells. The hair bundle is an apical specialization consisting of actin-filled membrane protrusions (called stereocilia) connected by tip links (TLs) that transfer the deflection force to gate the mechanotransduction channels. Here, we identified the hearing loss-associated *Loxhd1/DFNB77* gene as being required for the mechanotransduction process.

## Introduction

Hair cells are inner ear mechanosensors that detect the forces generated by sound and head motions. Hair-cell apical membrane protrusions, called stereocilia, are filled with F-actin and are organized into rows of increasing height (Tilney and Saunders, 1983). The taller stereocilia convey displacement forces to the tips of the shorter stereocilia [which host mechanotransducer (MET) channels] via an external filament called the tip link (TL; Pickles et al., 1984; Assad et al., 1991). MET channels are gated by the TL and by additional elements arranged mechanically in series with it, such as the TL insertion protein complexes, MET channel accessory subunits, and, potentially, cytoskeletal and stereociliary membrane connectors (Howard and Hudspeth, 1988; Bartsch et al., 2019; Ó Maoiléidigh and Ricci, 2019). All known molecular MET machinery components are clustered at one of the two TL insertion points: at the upper TL (UTL), Harmonin, MYO7A, and USH1G maintain TL tension (Kros et al., 2002; Michalski et al., 2009; Grillet et al., 2009a; Caberlotto et al., 2011; Grati and Kachar, 2011; Li et al., 2020); whereas at the lower TL (LTL), LHFPL5, TMIE, CIB2, TMC2, and TMC1 form the MET channel complex (Kawashima et al., 2011; Xiong et al., 2012; Zhao et al., 2014; Kurima et al., 2015; Giese et al., 2017; Cunningham et al., 2020), among which TMC1 and TMC2 may contribute to the pore (Pan et al., 2018; Jia et al., 2020). All of these components are required from the onset of cochlear hair-cell mechanotransduction at postnatal day 1 (P1), with the exception of TMC1, which is only required after P7 because of earlier functional compensation by TMC2 (Kawashima et al., 2011; Kim and Fettiplace, 2013; Pan et al., 2013; Beurg et al., 2018). Additional proteins are thought to be necessary for the mechanotransduction process, by contributing to the complexes, targeting MET components to the hair bundle (Cunningham et al., 2017), or regulating MET activity in the hair bundle. Defects in candidate proteins for such functions are expected to cause hearing loss.

Mutations in *LOXHD1* cause DFNB77, a human nonsyndromic recessive form of hearing loss (Grillet et al., 2009b) that can manifest at birth or later in life and shows various degrees of progression, preferentially affecting the high and middle hearing frequencies (Grillet et al., 2009b; Edvardson et al., 2011; Eppsteiner et al., 2012; Vozzi et al., 2014; Atik et al., 2015; Mori et al., 2015; Minami et al., 2016; Wesdorp et al., 2018; Maekawa et al., 2019). *Loxhd1* encodes a protein consisting of 15 polycystin lipoxygenase α-toxin (PLAT) repeats (Grillet et al., 2009b). PLAT repeats are part of the PLAT domain family (conserved protein domain #cd00113). PLAT domains allow interactions with lipids and proteins and form 120–150 amino acids β-barrel sandwiches with highly conserved residues (Bateman and Sandford, 1999).

In the inner ear, the *Loxhd1* mRNA is selectively expressed in hair cells; moreover, the protein encoded by it is localized in the hair bundle and is expressed at low levels at P2, followed by an increase to high levels at P10 (Grillet et al., 2009b). *Loxhd1^Sba/Sba^* mice, which carry a missense mutation in the PLAT10 repeat (I1342N), exhibit hearing loss at 3 weeks of age (Schwander et al., 2007; Grillet et al., 2009b). Although these results indicate that hair-cell function is compromised in these mice, the molecular role of LOXHD1 remains unknown.

As the *Loxhd1^Sba/Sba^* outer hair cells (OHCs) were able to mechanotransduce at P7 (Xiong et al., 2012), we hypothesized that the onset of a MET phenotype could occur later. Using two mouse mutants of LOXHD1-PLAT10, we demonstrated here that inner hair cell (IHC) mechanotransduction currents were drastically reduced at P11, in contrast to the wild-type (WT)-like MET currents observed at P7. Thus, our study has identified a critical step in hair-cell MET maturation. When this step was defective in our mutants, although the hair bundle structure was maintained and major components of the MET complex were present, MET activity was affected and hearing loss occurred.

## Materials and Methods

### 

#### Animal model and Loxhd1^Sba^ allele

The Administrative Panel on Laboratory Animal Care (APLAC) at Stanford University (APLAC protocols #28278 and #30305) approved all animal procedures. Mice of both sexes were used in all experiments. No sex-specific phenotype was observed in any of the mutants. WT or heterozygous littermates served as controls. The *Loxhd1^Sba^* strain was described previously (Schwander et al., 2007; Grillet et al., 2009b) and carries a missense mutation affecting the PLAT10 repeat (I1342N from the reference cDNA GenBank accession no. FJ750876 cloned from organ of Corti). The *Loxhd1^Sba^* mouse strain was provided by the Scripps Research Institute. Genotyping was performed by PCR around the missense mutation using the primers indicated below, followed by fragment purification and sequencing [Samba-S: GTGGTGCGCTGACTGGTATGTGG; Samba-AS: GCCCTTTTCCTGTGCCTGCTCAT, 466 bp; genotyping sequence: GTGGAAAT/ACTGGAA (T, WT allele; A, mutant allele)]. The *Loxhd1^Sba^* strain was produced and backcrossed onto the C56BL/6J background >20 times.

#### Generation of the Loxhd1^T1308X^allele

Targeting was conducted by homologous recombination in embryonic stem (ES) cells. A *Loxhd1^T1308X^* vector was designed by inserting a stop codon after the two first coding codons of exon 30, and the remaining sequence of exons 30 and 31 was removed (p.T1308X_ P1420del of the reference cDNA GenBank accession no. FJ750876) and replaced by the FRT-pGK-Neo-pA-FRT cassette. The homology arms were amplified from BAC DNA bMQ-372L7 (129S7/AB2.2; Source BioScience) using the following primers: 5′ arm, ClaI-SnaBI-5′-arm-S: AGATCGATTACGTAGCATCAGAGCAAAGCCAAGACCAC; with 5′ arm-STOP-EcoRV-AS: TTGATATCATCAGTCAGCCTGGAGGGTCCAAATATG; 3′ arm, SacII-MfeI-3′arm-S: GTCCGCGGCAATTGTTAGAAAATGAGCAGGCACAGG; with 3′ arm-PacI-SacII-AS: TACCGCGGTTAATTAATTCCCCAATGAGCAAGACAAA; followed by cloning into a pBluescript vector containing the FRT-pGK-Neo-FRT cassette. The targeting vector was electroporated into 129P2/OlaHsd-derived E14TG2a ES cells. ES clones were screened by PCR for the homologous integration of the 3′ arm (ES-screen-Neo-S: CTTGGCGGCGAATGGGCTGACC with ES-screen-outside-3′ arm-AS: GGGCCAGCACCTCTATTTTCTTGATGA). Positive clones were analyzed by Southern blotting using an internal probe. Two confirmed clones were injected into OLA129 blastocysts at the Scripps Research Institute transgenic facility. The resulting chimeras were mated to C57BL/6J females to obtain germline transmission. Heterozygous F1 mice were mated with B6.Cg-Tg(ACT-FLPe) mice (stock #005703, The Jackson Laboratory) to remove the pGK-Neo-cassette, and the resulting offspring were subsequently mated to C57BL/6J mice to remove the FLPe transgene, followed by backcrossing onto the C57BL/6J background >14 times. The *Loxhd1^T1308X^* strain was provided by the Scripps Research Institute. *Loxhd1^T1308X^* genotyping was performed by tail-DNA PCR using the following primers: Loxhd1-T1308X-S: GGTCCCCTGCCAAGCCCTCAT; with Loxhd1-T1308X-S: CAGGGTGAGGGCAGAGTTTAGGAC. The mutant allele produced a 457 bp band, while the WT allele produced a 787 bp band.

#### Quantitative real-time PCR and RT-PCR

Total RNA was extracted from cochlea using the NucleoSpin RNA XS Kit (catalog #740902, MACHEREY-NAGEL) and converted to cDNA by reverse transcription using Superscript III (Thermo Fisher Scientific). To quantify PLAT domain expression levels, equal amounts of cDNA were mixed with Maxima SYBR Green qPCR Master Mix (Thermo Fisher Scientific) and 300 nm sense and antisense primers specific for different PLAT domains, as follows: PLAT1-S: TACGAGGAGGAGCTGCTGAACTACGA; with PLAT1-AS: CATCCCCTGTGGCTGTGACC, PLAT5-S: GGAATGGCCCGGTATCGTGTGA; with PLAT5-AS: GGTCGGTGTTGTTCCTGCAGTTGTAAA; PLAT10-S: GCAAGTCAGAGAACCGCACCAACAAG; with PLAT10-AS: AAGACAAAGGCACGGCTATCAGT; PLAT15-S: AGGCTGGCTGGTGGAGAAGGTG; with PLAT15-AS: TGCCGCAGGAGAAGATTGTGG; GAPDH-S: CCACCCAGCCGAGAGGAATGA; with GAPDH-AS: GCAAACGGGAAGGAAATGAATGAA. Triplicate quantitative real-time PCRs were performed on P26 *Loxhd1^Sba/Sba^* (*n* = 6), *Loxhd1*^*Sba*/+^ (*n* = 4), *Loxhd1*^+/+^ (*n* = 4), *Loxhd1^T1308X/T1308X^* (*n* = 4), *Loxhd1*^*T1308X*/+^ (*n* = 3), and *Loxhd1*^+/+^ (*n* = 3) littermates. The relative expressions of PLAT-coding exons were calculated as 2^−ddCt^ by normalization against *Gapdh* and WT PLAT1 and plotted as whisker plots (±SEM). The relative values were compared using two-way ANOVA with the three genotypes as independent variables. When a genotype-specific difference was identified, the mean values were compared using Tukey's multiple-comparison test. RT-PCR was performed around exons 26/32 on P25 cochlear total RNA using a poly-dT primer for the single-stranded cDNA and the following primers: Ex26-S: GGCTTCCCGCTTCATTGTGG; with Ex32-AS: AGTGGCTGCCTCCTTGTGTTTC. The PCR products were gel purified, Sanger sequenced, and analyzed using LaserGene (DNASTAR) software.

#### Antibodies

The anti-PLAT11/12 antibody was previously validated *in vitro* on 293T cells by immunofluorescence and Western blotting (Grillet et al., 2009b). The anti-PLAT11/12 antibody corresponds to the polyclonal IgG fraction of a rabbit immunized against the following peptides: VTTGKHKEAATDSRAF and NGSTEEVQLDKKKARFEREQND. Attempts to affinity purify this antibody against the synthetic peptides or the *in vitro*-produced PLAT11/12 failed, as the eluted antibodies had lost their immunoreactivity on hair cells. Anti-PLAT11/12 was used at a 1:1000 dilution on whole-mount cochlea.

Other antibodies used here included the following: anti-Harmonin-H3 antibody (rabbit IgG affinity-purified against Harmonin-b-MBP, as described in Grillet et al., 2009a; a gift from U. Müller, John Hopkins University School of Medicine, USA), used at 1:300; and rabbit monoclonal anti-LHFPL5 antibody (catalog # ab192242, Abcam), used at 1:100. The Harmonin and the LHFPL5 antibodies were validated on knock-out mouse models (Grillet et al., 2009a; Li et al., 2019).

#### *In vitro* validation of anti-LOXHD1 antibody

293T cells (stock # CRL-3216, ATCC) were transfected with pcDNA3-mLOXHD1-Myc, which encodes the mouse full *Loxhd1* coding sequence (PLAT1 to PLAT15). The expression vector pcDNA3-mPKD1-PLAT-HA, which contains the PLAT domain of mouse PKD1, was transfected as a control to evaluate possible cross-reactions. Twenty-four hours after transfection, the cells were fixed with 4% PFA for 10 min at room temperature (RT) and then blocked and permeabilized with PBS containing 0.5% saponin and 4% BSA for 1 h at RT. The rabbit anti-PLAT11/12 (1:500) antibody was used for costaining with the mouse anti-Myc (1:4000; catalog #9B11, Cell Signaling Technology) or rat anti-HA (1:500; catalog #3F10, Roche) antibody. Alexa Fluor 488-conjugated donkey anti-rabbit (catalog #A-21206, Thermo Fisher Scientific), Alexa Fluor 568-conjugated goat anti-mouse (catalog #A-11019, Thermo Fisher Scientific), and Alexa Fluor 568-conjugated goat anti-rat (catalog #A-11077, Thermo Fisher Scientific) antibodies were used as secondary antibodies (1:500 dilution). The cell nuclei were stained with DAPI. The images were captured on an LSM 880 Confocal Microscope (Zeiss).

To generate pcDNA3-LOXHD1-Myc, the full *Loxhd1* coding sequence was cloned from P7 mouse organ of Corti using the following primers: NG-138, ATGATGGCCCAGAAGAAGAAGCGGAG; and NG-64, ACACCCTGCAGCAAGTCCCAACC; followed by reamplification using primers containing XhoI and EcoRI sites, for cloning into pcDNA3. A Myc tag was added using NEBuilder HiFi DNA assembly (New England Biolabs) with the following primers: PW-83, GAACAAAAACTCATCTCAGAAGAGGATCTTGAGAATTCCACCACACTG; and PW-84, TCTGAGATGAGTTTTTGTTCAACGGCCGCGACAGACGGGAAGAGCTC.

To generate pcDNA3-PKD1-PLAT-HA, a DNA fragment encoding the PLAT domain of PKD1 was cloned from P10 mouse cochlea cDNAs using the following primers: PW-284-CCCTCTAGACTCGAGACCATGATGTTCAAATATGAAATACTTGTTA; and PW-285, TCTGGAACATCGTATGGGTAAACGGCCGCAAGCACCTCCTTCTCCACTA. The pcDNA3 vector was amplified with the primers PW-85, TACCCATACGATGTTCCAGATTACGCTTGAGAATTCCACCACACTG; and PW-219, GGTCTCGAGTCTAGAGGG. An HA tag was included in the primers, and expression vector was constructed by assembling the two fragments using the NEBuilder HiFi DNA assembly (New England Biolabs).

#### Whole-mount immunostaining

The inner ears of P7 and P11 mice were dissected from temporal bones in washing buffer (WB; 0.05 mm HEPES buffer, pH 7.2, 10 mm CaCl_2_, 5 mm MgCl_2_, and 0.9% NaCl) at RT and were placed in a dish containing fixative (4% PFA in WB). A hole was poked in the cochlear apex using a needle, fixative was perfused slowly through the round window, and the sample was fixed for 30 min at RT. Samples were transferred to WB and the cochlear shell, stria vascularis, Reissner's membrane, and tectorial membrane were removed. The samples were washed three times, 5 min/wash, with WB containing no calcium or magnesium (0.05 mm HEPES buffer, pH 7.2, and 0.9% NaCl) and permeabilized at RT in WB containing 0.02% Triton X-100 for 10 min (for anti-PLAT11/12 antibodies), or 0.5% Triton X-100 for 20 min with 4% BSA Fraction V (for anti-Harmonin and anti-LHFPL5). They were then blocked with 4% BSA fraction V in WB for 1 h at RT. The samples were subsequently incubated with the primary antibodies diluted in 1% BSA fraction V in WB containing 0.02% Tween-20 overnight at 4°C with gentle rocking. After three washes with WB, 5 min/wash, the secondary antibody anti-rabbit Alexa Fluor-564 or Alexa Fluor-488 (Fisher) at 1:500 and Phalloidin-488 or Phalloidin-568 (Fisher) at 1:300 were added to the samples, which were then incubated at RT with gentle rocking for 1 h. After 3 × 5 min washes with WB, samples were mounted using a slide and coverslip with ProlongGold antifade medium (Thermo Fisher Scientific). Samples were imaged using a Zeiss LSM880 microscope in Airyscan mode and a 63× Plan-Apochromat 1.4 oil lens. Staining was repeated on more than three animals per condition.

#### Quantification of LHFPL5 and Harmonin at the TL ends

Each immunofluorescence staining was replicated at least three times. The percentage of stereocilia with either Harmonin puncta at the UTL insertion point in the tall row or LHFPL5 puncta at the tip of the second row (LTL insertion point) was determined. If the region of interest was not visible, the stereocilium was disregarded. The *p* values were calculated using Mann−Whitney tests.

#### Auditory measurements

We measured auditory brainstem responses (ABRs), distortion products of otoacoustic emissions (DPOAEs), and cochlear microphonics (CM) in anesthetized mice by administering 100 mg/kg ketamine and 10 mg/kg xylazine intraperitoneally, and their body temperature was held constant at 37°C until they had recovered fully. Sound stimuli were generated digitally by a self-written software (Oghalai, 2004) in MATLAB (version 7.0; MathWorks) that controlled a custom-built acoustic system using a digital-to-analog converter (catalog #NI BNC-2090A, National Instruments), a sound amplifier (catalog #SA1, TDT), and two high-frequency speakers (catalog #MF1, TDT). The speakers were connected to an ear bar and calibrated in the ear canal before each experiment using a probe-tip microphone (microphone type 4182, NEXUS conditioning amplifier, Brüel and Kjær). ABRs were recorded by placing three needle electrodes subcutaneously at the vertex, below the left ear, and a ground electrode close to the tail. The signals were amplified 10,000 times using a biological amplifier (catalog #DP-311, Warner Instruments) digitized at 10 kHz, and digitally bandpass filtered from 300 to 3000 Hz. The stimulus for eliciting the ABR was a 5 ms sine wave tone pip with cos^2^ envelope rise and fall times of 0.5 ms. The repetition time was 50 ms, and 260 trials were averaged. At each frequency, the peak-to-peak voltages of ABR signals at stimulus intensities ranging from 10 to 80 dB sound pressure levels (SPLs) were measured in 10 dB steps and fitted and interpolated to find thresholds that were 5 SDs above the noise floor. For statistical purposes, we defined the threshold as 80 dB SPL, if no ABR was detected.

For the stimulation of DPOAEs, two sine wave tones of equal intensity (l1 = l2) and a duration of 1 s were presented to the ear. The tones ranged from 20 to 80 dB SPL attenuated in 10 dB increments, and the two frequencies (f2 = 1.2f1) ranged from f2 with 4–32 kHz. The acoustic signal detected by the microphone in the ear bar was sampled at 200 kHz, and the magnitude of the cubic distortion product (2f1 – f2) was determined by fast Fourier transform. The noise floor was determined by averaging 20 adjacent frequency bins surrounding the distortion product frequency. DPOAE thresholds were reached when the signal was >3 SDs above the noise floor.

For CM, the head of the anesthetized animal was secured in a holder. The left tympanic bulla was surgically uncovered and opened to expose the round window. The CM was measured from the ball-ended tip of a Teflon-coated silver wire (diameter, 0.003; A-M Systems) that was advanced onto the round window membrane with a micromanipulator. The signal was referenced to a silver wire inserted under the skin near the vertex of the skull. The ground electrode was placed in the hindleg. A bioamplifier was used (DP-311 differential amplifier, Warner Instruments) to amplify the signals 100 times, which were then bandpass filtered between 1 Hz and 10 kHz. The sound stimuli were 20 ms, 6 kHz tones with an intensity ranging from 10 to 100 dB in 5 dB steps. The stimuli were synthesized in a software and output by a speaker (model #MDR EX37B, Sony) inserted into the ear canal. We calibrated the intensity using a probe tip microphone in the ear bar, as described previously (Xia et al., 2010). The CM signal measured by the bioamplifier was digitized at 1 MHz, and the magnitude of the response at 6 kHz was determined by fast Fourier transform. All stimulus harmonics and noise at all other frequencies were ≥50 dB below the primary signal at all stimulus intensities. The control animals used in this experiment were heterozygous littermates that gave results similar to those of age-matched WT mice.

#### Electrophysiology

Recordings were performed on the apical cochlear turn, which corresponded to a 6−10 kHz frequency in the adult mouse.

Whole-cell patch-clamp recordings were performed at RT (19–22°C) with borosilicate patch pipettes with a resistance of 2.5–3.5 MΩ. MET currents were low-pass filtered at 100 kHz, measured with an Axopatch 200B patch-clamp amplifier, digitized with a daq3000 instrument (IOtech) at 500 kHz, and recorded using jClamp (SciSoft). MET currents were filtered offline at 10 kHz for further visualization and analysis. Voltages were adjusted offline for the liquid junction potential, and cells were held at −84 mV. Cells with >80 pA of leak current were discarded. The uncompensated series resistance was between 6 and 10 MΩ. The organ of Corti was perfused with external solution via an apical perfusion pipette placed 20–30 µm away from the cell that was being patched. Recordings were visualized and analyzed using jClamp (SciSoft) and OriginPro 2018 (OriginLab). Graphs were generated using OriginPro 2018 (OriginLab) and Adobe Illustrator (CS4, Adobe). For fluid jet stimulation of hair bundles, we used a piezoelectric disk bender driving the fluid stimulation through a pipette with a diameter of 8–12 µm. The voltage stimulus that was used to drive the disk bender was filtered at 1 kHz with an 8-pole Bessel filter (Frequency Devices). Stiff-probe recordings were conducted as described previously (Effertz et al., 2017). The tissue was perfused with extracellular solution containing the following: 145 mm NaCl, 2 mm KCl, 2 mm CaCl_2_, 1 mm MgCl_2_, 10 mm HEPES, 6 mm glucose, 2 mm pyruvate, 2 mm ascorbic acid, and 2 mm creatine monohydrate. The pH of the external solution was adjusted to 7.4 by the addition of NaOH, and the osmolality ranged from 304 to 308 mOsm. The internal patch solution contained the following: 116 mm CsCl, 3.5 mm MgCl_2_, 3.5 mm ATP, 5 mm creatine phosphate, 0.1 mm tetracesium BAPTA, 10 mm HEPES, and 20 mm ascorbic acid. The internal solutions were adjusted to pH 7.2 with CsOH, and the osmolality ranged from 282 to 286 mOsm. All recorded currents are included in the summary plots.

#### Scanning electron microscopy

Inner ears were isolated in WB (0.05 mm HEPES buffer, pH 7.2, 10 mm CaCl_2_, 5 mm MgCl_2_, and 0.9% NaCl) and fixed in 4% PFA in 0.05 mm HEPES buffer, pH 7.2, 10 mm CaCl_2_, 5 mm MgCl_2_, and 0.9% NaCl for 30 min at RT. The inner ears were then dissected to remove the stria vascularis and Reissner's and tectorial membranes. The samples were refixed in 2.5% glutaraldehyde and 4% PFA in 0.05 mm HEPES buffer, pH 7.2, 10 mm CaCl_2_, 5 mm MgCl_2_, and 0.9% NaCl overnight at 4°C, then washed, dehydrated in ethanol (30%, 75%, 100%, and 100%, 5 min incubations), and processed to the critical drying point using Autosamdri-815A (Tousimis). Cochleae were mounted on studs using silver paint and coated with 5 nm of iridium (sputter coater EMS150TS, Electron Microscopy Sciences). Samples were imaged at 5 kV on an FEI Magellan 400 XHR Field Emission Scanning Electron Microscope at the Stanford Nano Shared Facilities. The microscope is periodically calibrated for measurements using a SIRA-type calibration specimen for ultra-high-resolution modes with ≤2% error between 50 and 350k× magnification at our imaging settings.

#### Quantification of TLs

Scanning electron microscopy (EM) pictures of apical turn IHCs were deidentified from genotype information before counting. TLs that connected the second row of stereocilia to the first (tallest) row were counted in P7 and P11 mice. Only second-row stereocilia that were designated as paired with their corresponding first-row stereocilia (based on their relative position from the center of the hair bundle and the distance between the two stereocilia) were used for quantification. If it was unclear whether a second-row stereocilium was paired, it was not included in the statistics. Second-row stereocilia that appeared to be paired, but with tips that were not in the field of view of the image (e.g., blocked by another object), were also discounted. For all paired stereocilia, only visible links that connected stereocilia in the direction of stimulation were tallied as TLs. Paired stereocilia with no visible link were considered to have no TL. The *p* values were calculated using Mann−Whitney tests.

#### Determination of the height of stereociliary rows

To compare stereociliary heights between control (WT and heterozygous littermates) and mutant animals, we chose to maximize sampling by quantifying 12–21 apical IHCs at P7 and P11. From scanning EM pictures almost orthogonal to the face of the hair bundle, we measured the height of each staircase step, up to the fourth row, using Fiji software. If more rows were present, the entire height of row 4 was determined. As an approximation of the absolute height, we summed the corresponding steps, likely leading to a slight overestimation of height across all measurements. Five to 12 stereociliary columns were measured per cell and plotted as the average per cell. Unpaired nonparametric Mann−Whitney scores were obtained to compare stereocilia height from IHCs of different genotypes.

#### Experimental design and statistical analyses

Data collection was performed by experimentalists who were blinded to the genotypes of the animals. Genotype was revealed during the analytical phase to ensure that appropriate *n* values were obtained between groups. Although gender was noted, no gender differences were observed between groups, and data are presented with genders combined. The statistical analyses are described in the Results section and were performed using the Origin (OriginLab) or Prism7 (GraphPad) software. In all cases, *p* < 0.05 was considered significant.

##### Qualitative PCR.

Qualitative PCR results are described in Materials and Methods.

##### ABR and DPOAE.

ABR and DPOAE thresholds were determined at 4, 6, 8, 11, 24, and 32 kHz for P21 and P60 *Loxhd1*^*T1308X*/+^ littermate animals and *Loxhd1^Sba/Sba^* animals for comparison, as follows: P21: *Loxhd1^T1308X/T1308X^* (*n* = 13), *Loxhd1*^*T1308X*/+^ (*n* = 16), *Loxhd1*^+/+^ (*n* = 8), and *Loxhd1^Sba/Sba^* (*n* = 9); and P60: *Loxhd1^T1308X/T1308X^* (*n* = 9), *Loxhd1*^*T1308X*/+^ (*n* = 8), *Loxhd1*^+/+^ (*n* = 4), and *Loxhd1^Sba/Sba^* (*n* = 9). In *Loxhd1^T1308X/T1308X^* animals, we observed heterogeneity in the responses to 11 kHz sound stimulation at 80 dB, which was the highest sound intensity tested: some animals responded while others did not. The number of animals in each responder group are indicated. The mean threshold values are indicated in decibels SPL and SDs.

##### CM.

The magnitude of the CM signal (peak-to-peak amplitude in microvolts ± SD) was measured in P26 *Loxhd1^T1308X/T1308X^* (*n* = 4), *Loxhd1*^*T1308X*/+^ (*n* = 4), *Loxhd1^Sba/Sba^* (*n* = 3), and *Loxhd1*^*Sba*/+^ (*n* = 4) littermates. Heterozygous littermates gave similar results to those of age-matched WT animals.

##### Single-cell electrophysiology recordings.

We used whole-cell voltage-clamp and hair bundle fluid jet mechanical stimulation in apical IHCs between P7 and P11 on littermate control animals (pooled P7 animals: three *Loxhd1*^*T1308X*/+^, two *Loxhd1*^*Sba*/+^, and one WT; P11 pooled animals: three *Loxhd1*^*T1308X*/+^, one *Loxhd1*^*Sba*/+^, and two WT animals), *Loxhd1^T1308X/T1308X^* mice (*n* = 4), and *Loxhd1^Sba/Sba^* mice (*n* = 4). Similar recordings were performed on P9 IHCs using a stiff probe on littermates [*Loxhd1*^*T1308X*/+^ (*n* = 15), *Loxhd1^T1308X/T1308X^* (*n* = 5), *Loxhd1*^*Sba*/+^ (*n* = 6), and *Loxhd1^T1308X/T1308X^* (*n* = 11)]. All recorded currents are included in the summary plots. Student's *t* tests were performed to compare current characteristics across genotypes.

Measurement of the heights of stereocilia, quantification of TLs, and quantification of LHFPL5 and USH1C at the TL ends are described in Materials and Methods.

## Results

### LOXHD1 expression in cochlear hair bundles increased from P2 to P7 to P11

**Figure 1.**
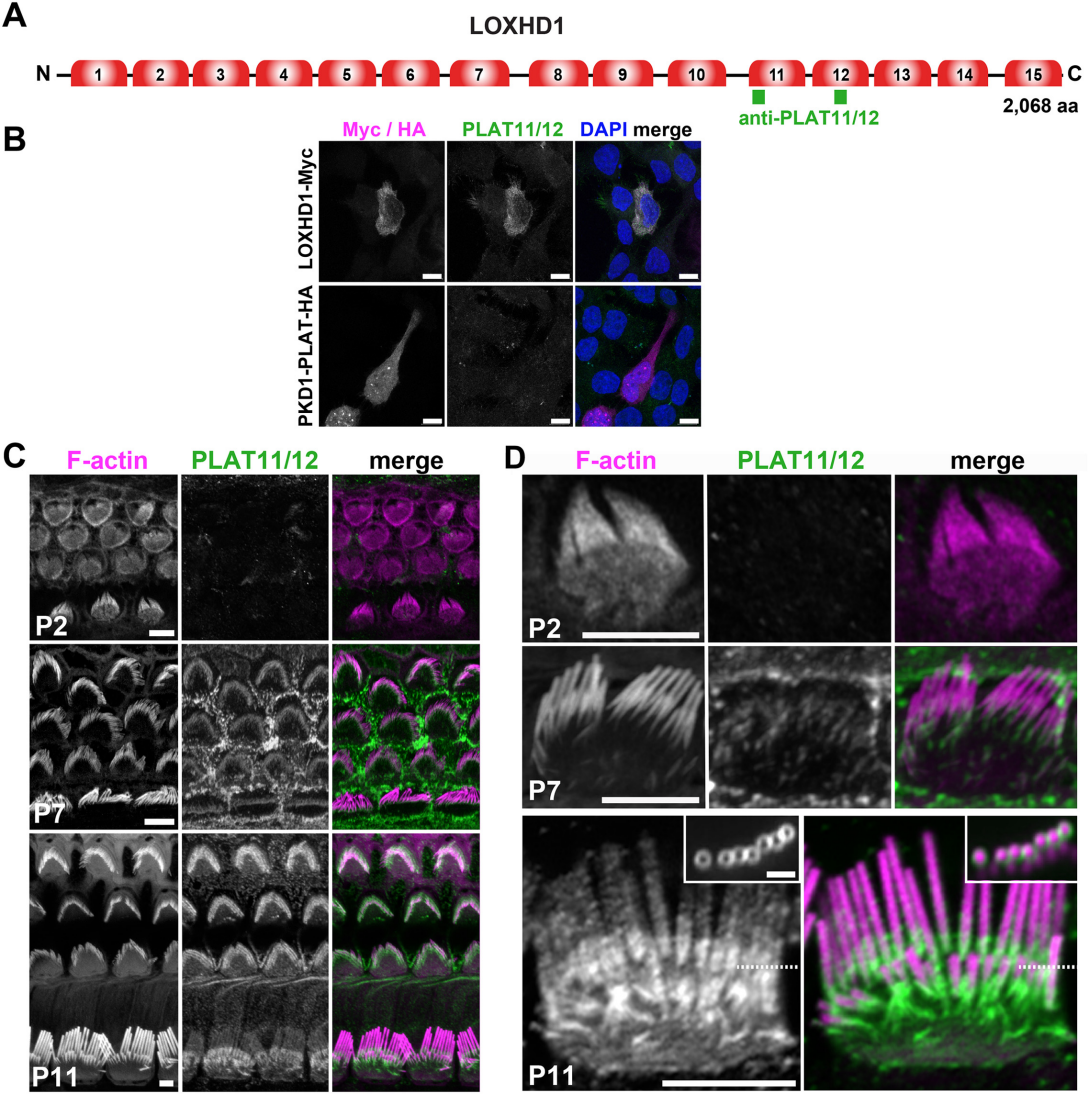
LOXHD1 expression in cochlear hair bundles increased from P2 to P7 to P11. ***A***, The LOXHD1 protein contains 15 PLAT domains predicted from cDNA FJ750876 cloned from organ of Corti. The locations of the peptides used to produce the anti-PLAT11/12 antibodies are indicated by the green boxes. ***B***, The anti-PLAT11/12 antibodies specifically recognized mLOXHD1-myc overexpressed in 293T cells for 24 h, and not mPKD1-PLAT-HA. Scale bars, 10 µm. ***C***, Immunofluorescence using the anti-PLAT11/12 antibodies on apical turns of the organ of Corti at P2, P7, and P11. Phalloidin was used to stain the F-actin-rich stereocilia core. Scale bars, 5 µm. ***D***, Apical IHC staining with the anti-PLAT11/12 antibody at P2, P7, and P11. Scale bars: 5 µm. Inset, A confocal section of the hair bundle across the tall and middle row (dashed line for position) shows a ring of signal around the actin core. Scale bar, inset in P11, 1 µm.

To gain new insights into the function of LOXHD1 in hair cells, we revisited its expression onset in the hair bundle (Grillet et al., 2009b). We used the antibody against PLAT repeats 11 and 12 reported in a previous study (here referred to as anti-PLAT11/12), which showed *in vitro* specificity, detecting a LOXHD1–Myc fusion construct overexpressed in 293T cells, but not the related PKD1 PLAT–HA fusion (Fig. 1*B*). We then stained the cochlear apical turn at different ages to localize the endogenous LOXHD1. At P2, anti-PLAT11/12 staining was restricted to weak labeling at the junctions between hair cells and supporting cells (Fig. 1*C*). Some hair bundle staining was detected at P7, together with some staining at the supporting cell apical surfaces. The latter is likely to be nonspecific because LOXHD1 mRNA was detectable only in the hair cells (Grillet et al., 2009b). At P11, stronger staining appeared in both OHC and IHC hair bundles. At P7, the anti-PLAT11/12 signal was restricted to the base of the stereocilia; however, at P11, the signal could be found along the entire length of the stereocilia, with an enrichment at the basal portion (Fig. 1*D*). The smallest rows appeared to be entirely stained. In optical sections of the stereocilia, anti-PLAT11/12 staining formed a ring around the actin core (Fig. 1*D*, inset). Overall, we conclude that LOXHD1 PLATs 11 and 12 are expressed gradually in cochlear hair-cell hair bundles, with an increase from an intermediate level to a high level occurring between P7 and P11.

### The nonsense mutation in *Loxhd1^T1308X/T1308X^* mice induces nonsense-associated altered splicing

**Figure 2.**
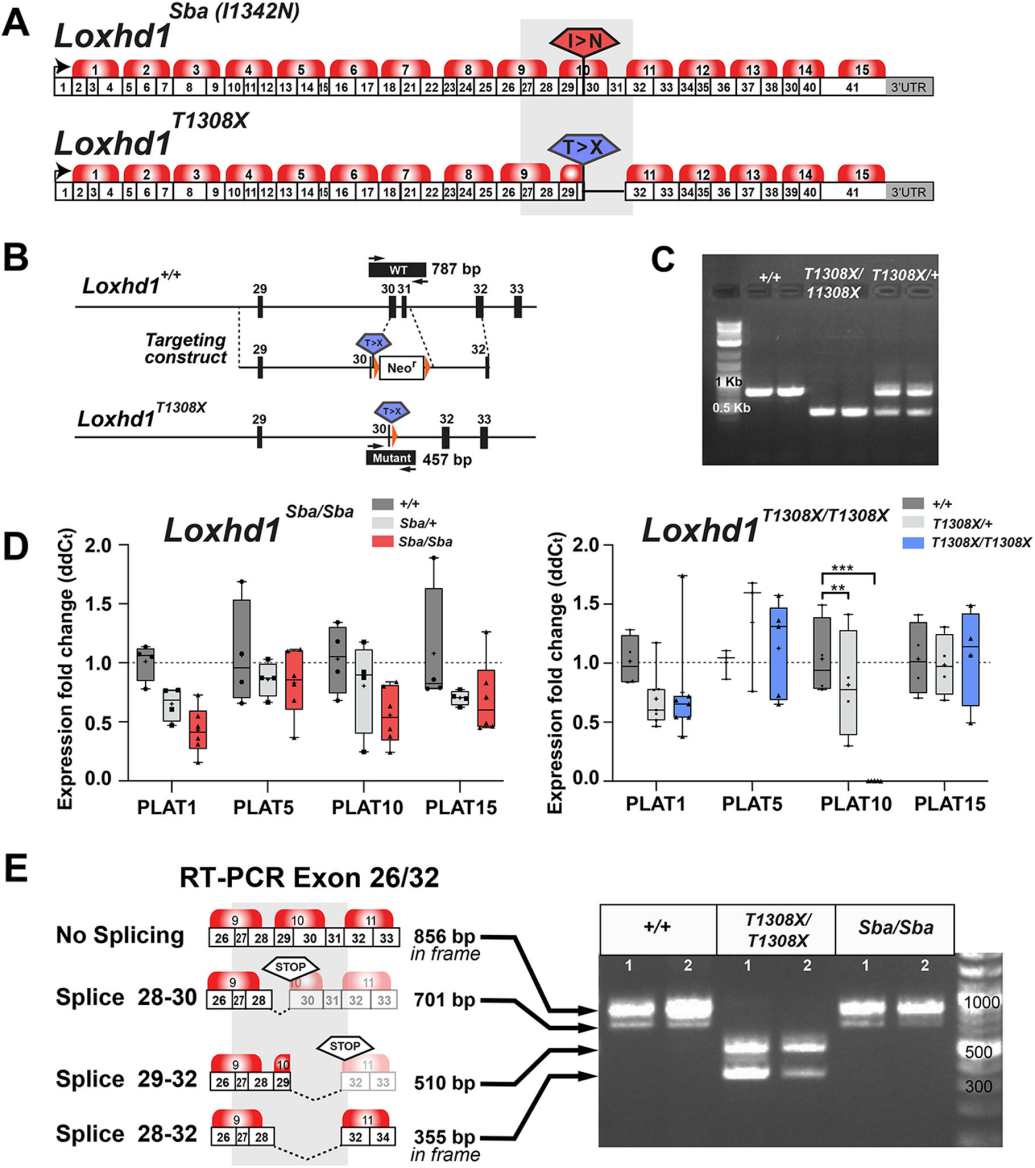
The nonsense mutation in *Loxhd1^T1308X^* induces NAS. ***A***, Representation of the mouse *Loxhd1^Sba^* (I1342N) and *Loxhd1^T1308X^* alleles (p.T1308X_ P1420del) on the reference cDNA GenBank accession number FJ750876 cloned from organ of Corti. ***B***, *Loxhd1^T1308X^* was produced by homologous recombination in embryonic stem cells. A nonsense codon was inserted after the two first codons of exon 30, and the downstream sequence until exon 31 was replaced with an FRT-pGK-Neo-pA-FRT cassette. After germline transmission, the selection cassette was removed *in vivo* by breeding the F1 with B6.Cg-Tg(ACT-FLPe) mice and backcrossing them over 14 generations onto a C57BL/6J background. The position of the PCR primers used for genotyping is indicated. ***C***, PCR genotyping at the *Loxhd1^T1308X^* allele produces a WT band at 787 bp and a mutant band at 457 bp. ***D***, The presence of a premature termination codon (PTC) in *Loxhd1^T1308X/T1308X^* did not induce nonsense-mediated mRNA decay. Quantitative real-time PCR along the *Loxhd1* cDNA corresponding to different PLAT repeats on P25 cochlear total RNA from *Loxhd1*^+/+^, *Loxhd1^T1308X/T1308X^*, and *Loxhd1^Sba/Sba^* animals. The relative PLAT-coding exon expression was calculated as 2^−ddCt^ (±SEM) with normalization against *Gapdh* and WT PLAT1. The individual expression fold change is indicated on a whisker plot, together with the median (bar) and average (cross sign) values, and was compared using two-way ANOVA and Tukey's *post hoc* multiple-comparison test from triplicates (to minimize the technical error) on a minimum of three animals stemming from three different litters per genotype (***p < 0.001, **p < 0.01). ***E***, Reverse transcription PCR on P25 total cochlear RNA from two animals per genotype. The RT-PCR bands were sequenced and the composition of the exons were determined by alignment against the reference sequence FJ750876. In WT and *Loxhd1^Sba/Sba^* animals, in addition to the full *Loxhd1* transcript, alternative splicing generated a PTC-containing isoform that was predicted to encode a protein ending after the PLAT9 repeat. In *Loxhd1^T1308X/T1308X^* animals, a splice variant led to a PTC, but, surprisingly, splicing also produced a transcript lacking exons 28–32 that still maintains the *Loxhd1* open reading frame.

The vast majority of the pathologic mutations in LOXHD1/DFNB77 patients correspond to nonsense mutations (Azaiez et al., 2018). The only mouse mutant previously available, *Loxhd1^Sba^*, carries a missense mutation affecting the PLAT10 repeat (I1342N), but otherwise produces a full-length protein (Fig. 2*A*; Schwander et al., 2007; Grillet et al., 2009b). To determine whether the effect of a nonsense mutation on hair cells was more severe than that of a missense mutation, we generated a novel *Loxhd1* mutant allele. We inserted a nonsense mutation in the PLAT10 coding sequence and deleted the next two coding exons (Fig. 2*A*,*B*). This approach was expected to truncate the protein at the center of the PLAT10 repeat and to provide an *in vivo* control for the anti-PLAT11/12 antibody. The *Loxhd1^T1308X^* allele (p.T1308X_ P1420del) was produced by homologous recombination in ES cells and injected into blastocysts to generate chimera (Fig. 2*B*,*C*; Materials and Methods). After removing the selection cassette using an FLPe deleter mouse, the allele was backcrossed over 14 generations onto the C57BL/6J background and produced genotypes in Mendelian ratios.

**Figure 3.**
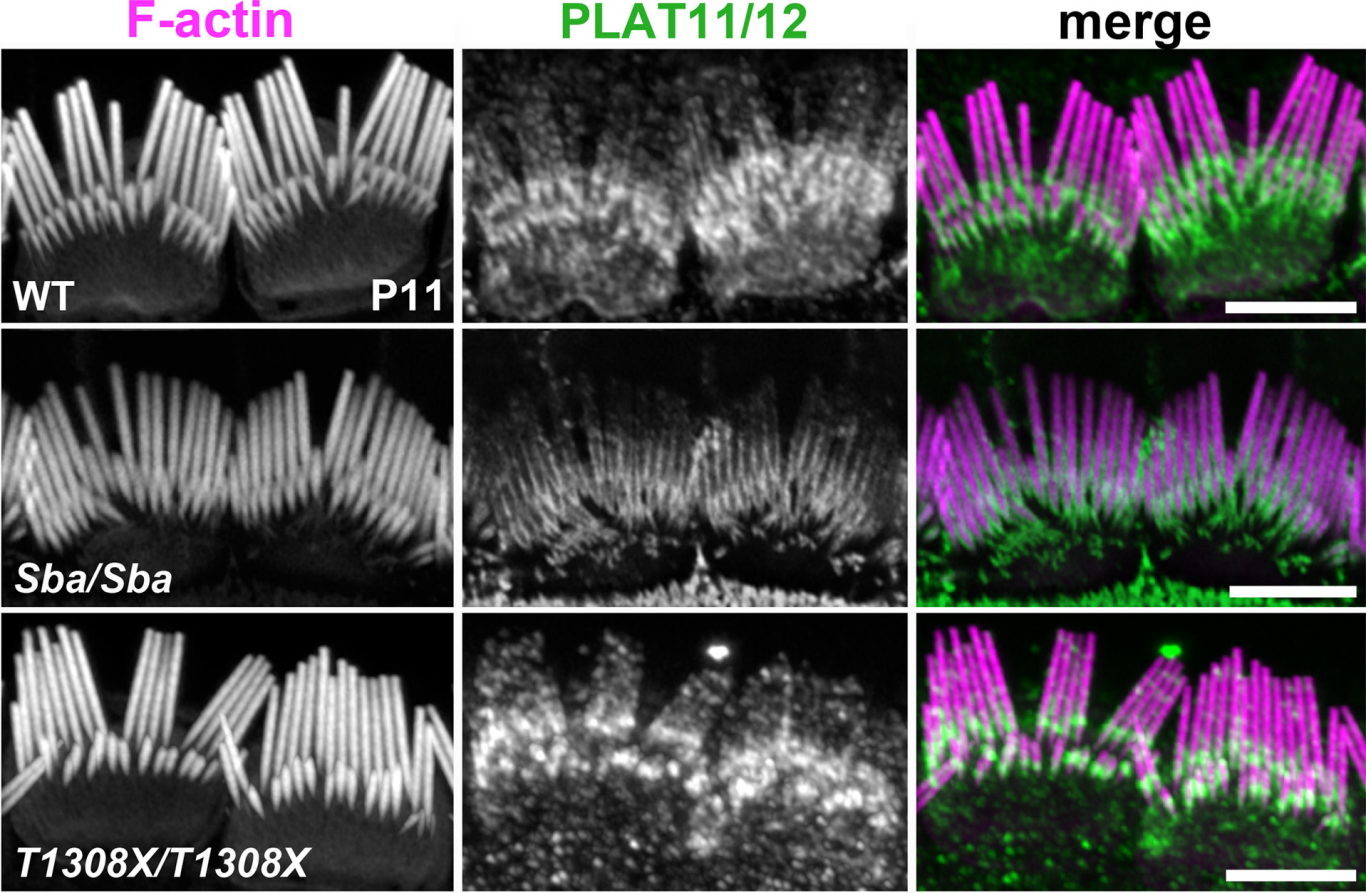
LOXHD1-PLAT11/12 signal is maintained in P11 IHC hair bundles of *Loxhd1^Sba/Sba^* and *Loxhd1^T1308X/T1308X^* mutants. Immunofluorescence staining of P11 IHCs using the anti-PLAT11/12 antibody shows hair bundle staining in *Loxhd1*^+/+^, *Loxhd1^T1308X/T1308X^*, and *Loxhd1^Sba/Sba^* samples. Scale bars, 5 µm.

We evaluated whether the premature termination codon in the *Loxhd1^T1308X^* allele triggered nonsense-mediated mRNA decay. The relative *Loxhd1* expression values were calculated as 2^−ddCt^ (±SEM) using *Gapdh* for normalization and the WT PLAT1 expression level as a reference for the other PLAT domains. The values were compared using two-way ANOVA and Tukey's *post hoc* multiple-comparison test (95% CI) from triplicates (to minimize technical errors) on a minimum of three animals stemming from different litters for each genotype. The premature termination codon of the *Loxhd1^T1308X^* allele did not trigger nonsense-mediated mRNA decay, as quantitative PCR at different *Loxhd1* mRNA positions revealed no significant differences between genotypes, other than the expected PLAT10 deleted area (Fig. 2*D*). Rather, we observed alternative splicing around the mutated area that rescued the *Loxhd1* open reading frame in *Loxhd1^T1308X/T1308^*, which was not observed in WT or missense *Loxhd1^Sba/Sba^* mice (Fig. 2*E*). The mRNA surveillance mechanism called “NAS” (nonsense-associated altered splicing; Valentine, 1998; Liu et al., 2001; Wang et al., 2002) presumably allows HCs to produce both a truncated form of LOXHD1 that ends in PLAT10 and an isoform that lacks PLAT10 but contains the remaining protein sequence. In accordance with the mRNA analysis, the anti-PLAT11/12 antibody stained the P11 IHC hair bundles of both *Loxhd1^Sba/Sba^* and *Loxhd1^T1308X/T1308X^* mutants (Fig. 3). In conclusion, the *Loxhd1^T1308X^* allele did not serve as a good control for our PLAT11/12 antibody because endogenous splicing rescued the coding sequence. Nevertheless, it will allow us to compare the effects of nonsense mutations on hearing and hair-cell function versus the corresponding effects of missense mutations where the relative mutations affect the same *Loxhd1* exon.

### Nonsense and missense mutations in *Loxhd1* lead to hearing loss and cochlear microphonic defects

**Figure 4.**
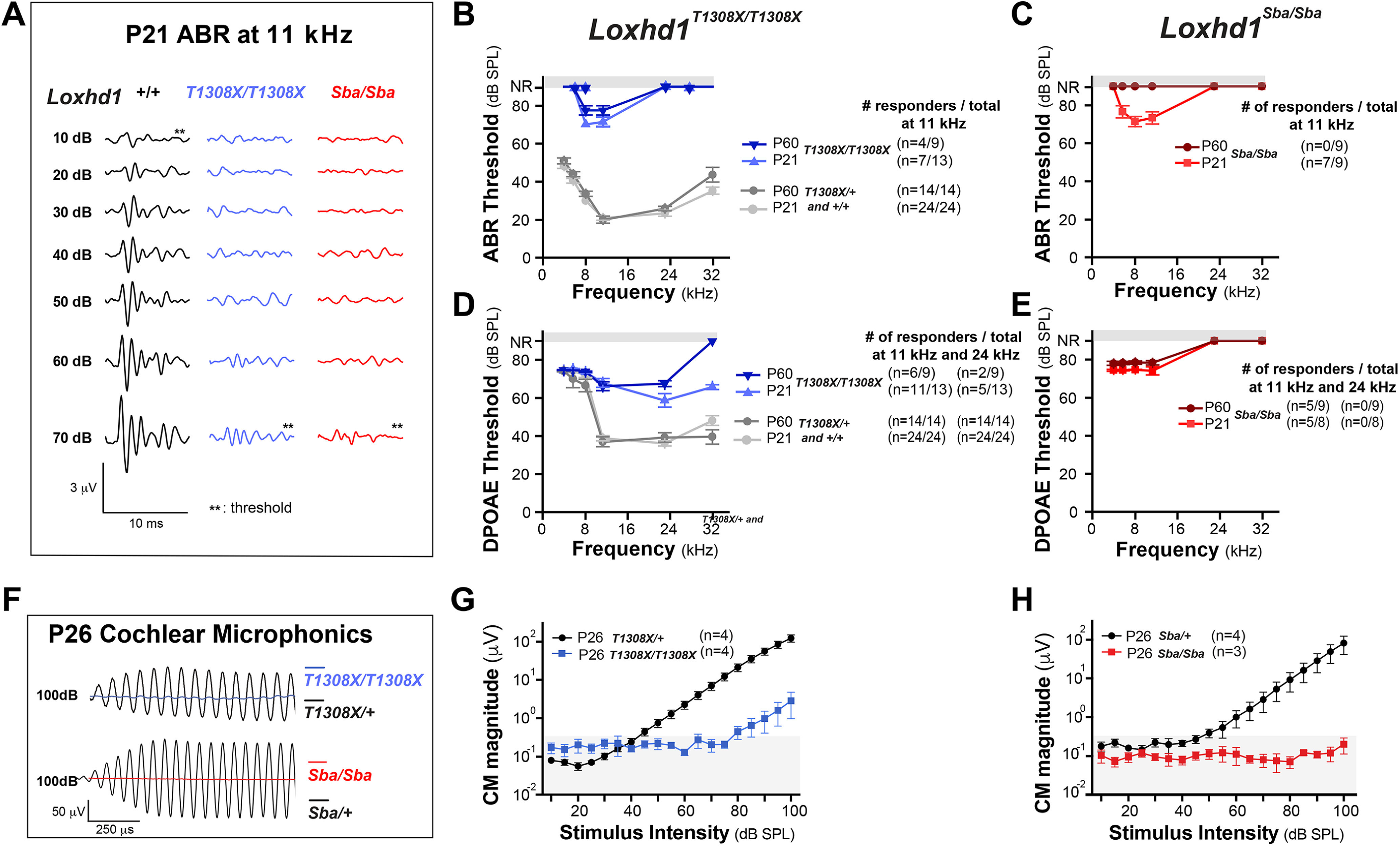
The *Loxhd1^T1308X/T1308X^* and *Loxhd1^Sba/Sba^* mutants exhibited severe hearing loss with residual responses. ***A***, ABR traces from *Loxhd1*^+/+^, *Loxhd1^T1308X/T1308X^*, and *Loxhd1^Sba/Sba^* P21 animals at 11 kHz. ABR thresholds are indicated by a double asterisk. ***B***, ***C***, ABR thresholds at 4, 6, 8, 11, 24, and 32 kHz for P21 and P60 *Loxhd1^T1308X/T1308X^* and *Loxhd1^Sba/Sba^* mutants compared with their heterozygous and wild-type littermates (pooled at a two-thirds/one-third ratio, respectively). In *Loxhd1^T1308X/T1308X^* animals, we observed heterogeneity in the responses to an 11 kHz sound stimulation at 80 dB, the highest sound intensity tested: some animals responded, while others did not. The numbers of each responder group are indicated (mean values are in dB, SPL ± SD). ***D***, ***E***, DPOAE thresholds under conditions equivalent to those in ***B*** and ***C***. The number of mice that responded at 11 and 24 kHz at 80 dB is indicated. ***F***, CM potential traces obtained at the round window in response to a 6 kHz sound at 100 dB SPL. ***G***, ***H***, The magnitude of the CM (peak-to-peak amplitude in µV ± SD) response was plotted against the sound intensity.

We then confirmed that the *Loxhd1^T1308X/T1308X^* animals exhibited hearing loss by measuring ABRs and DPOAEs (Fig. 4*A–E*). By P21, *Loxhd1^T1308X/T1308X^* animals exhibited thresholds that were elevated to at least 70 dB SPL for ABR and 60 dB SPL for DPOAE across frequencies, indicating severe hearing loss, and both IHCs and OHCs were affected, similar to the results obtained for our *Loxhd1^Sba/Sba^* mice. However, we noted that the *Loxhd1^T1308X/T1308X^* animals were less affected than the *Loxhd1^Sba/Sba^* animals, with residual responses being stable between P21 and P60 (Fig. 4*B–E*), together with reduced penetrance of hearing loss in the *Loxhd1^T1308X/T1308X^* animals. To determine whether the hair-cell dysfunction originated from a MET defect, we measured CM (Fig. 4*F–H*), which is a voltage measurement that reflects the summed currents across hair cells near the base of the cochlea on sound stimulation and is often used as an indirect *in vivo* readout of MET function (Cheatham et al., 2011). At P26, only a residual CM response was observed in *Loxhd1^T1308X/T1308X^* animals (>80 dB SPL; Fig. 4*G*), and no response was detected in *Loxhd1^Sba/Sba^* animals (Fig. 4*H*). In conclusion, both nonsense and missense mutations affecting *Loxhd1*-PLAT10 lead to hearing loss, potentially originating from a hair-cell MET deficit. Strikingly, we also demonstrated that the nonsense mutation followed by a short deletion yielded an attenuated hearing loss phenotype compared with the missense mutation, likely reflecting the selective occurrence of splicing rescue by NAS.

### *Loxhd1*-PLAT10 mutations affected IHC mechanotransduction at P11, but not at P7

**Figure 5.**
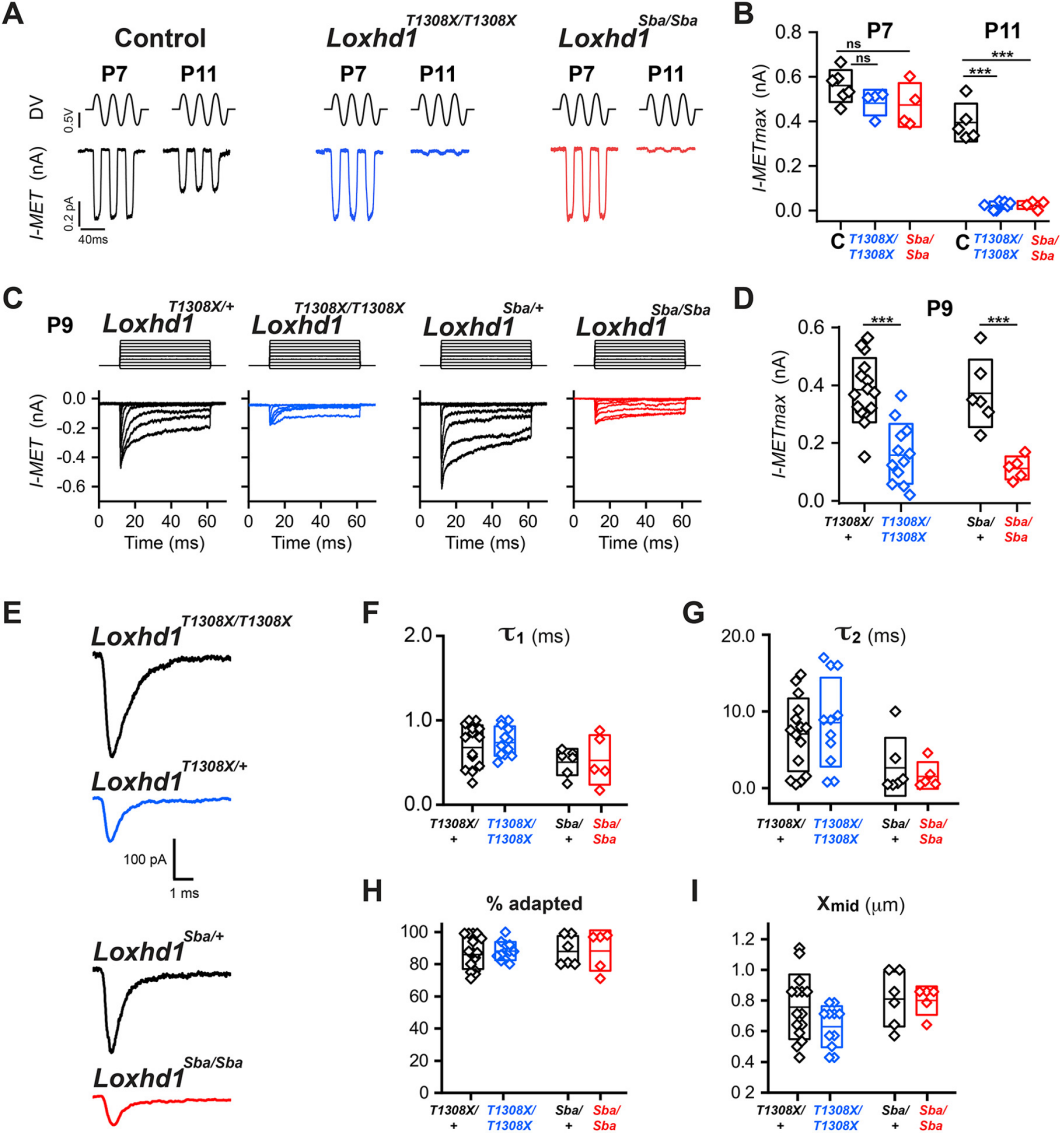
The *Loxhd1^T1308X/T1308X^* and *Loxhd1^Sba/Sba^* mutants have a MET current defect in IHCs after P7. ***A***, ***B***, Fluid jet mechanical stimulation of control (C; pooled heterozygous and wild-type littermates from both strains) IHCs produced inward currents that matured between P7 and P11. At P7, while the current amplitudes of *Loxhd1* mutant IHCs were similar to those of the control at P7, at P11 they became residual (75% of the cells) or absent. All recorded cells were included in the analysis. ***C***, ***D***, Stiff-probe mechanical stimulation of P9 IHCs from *Loxhd1*^*T1308X*/+^, *Loxhd1^T1308X/T1308X^*, *Loxhd1*^*Sba*/+^, and *Loxhd1^Sba/Sba^* animals. ***E***, Representative traces of P9 MET transduction current in littermates of the *Loxhd1^T1308X/T1308X^* and *Loxhd1^Sba/Sba^* mutants elicited by a 100 nm stiff-probe displacement. ***F–I***, The P9 time constants of fast adaptation (***F***), slow adaptation (***G***), percentage of adapted current (***H***), and half-activation displacement (***I***) were not significantly different between heterozygous and homozygous *Loxhd1^T1308X/T1308X^*. The box plots represent the mean (shown as a line) ± 1 SD. The values are indicated in Table 1.

To determine whether LOXHD1 is critical for the functional maturation of the hair bundle, we measured the MET currents *in vitro*. We used whole-cell voltage clamping and hair bundle fluid jet mechanical stimulation in apical IHCs between P7 and P11 (Fig. 5*A*,*B*, Table 1). In littermate control animals (pooled P7 animals: three *Loxhd1*^*T1308X*/+^, two *Loxhd1*^*Sba*/+^, and one WT; pooled P11 animals: three *Loxhd1*^*T1308X*/+^, one *Loxhd1*^*Sba*/+^, and two WT), we detected maximum MET current amplitudes (MET_max_) of 558 ± 72 pA (±SD) at P7 and 395 ± 85 pA at P11 (*n* = 5, *p* < 0.01, *t* test). This 29% MET_max_ reduction observed between P7 and P11 was in accordance with the findings of a previous report (Corns et al., 2018). At P7, the MET_max_ of *Loxhd1^T1308X/T1308X^* and *Loxhd1^Sba/Sba^* animals was similar to that of controls (484 ± 57 pA, *n* = 4, for *Loxhd1^T1308X/T1308X^*; 473 ± 98 pA, *n* = 4, for *Loxhd1^Sba/Sba^*; both n.s., *t* test). However, at P11, the MET_max_ was drastically reduced (95%) to 24 ± 16 pA (*n* = 8, *p* < 0.0001, *t* test) in *Loxhd1^T1308X/T1308X^* and to 25 ± 18 pA in *Loxhd1^Sba/Sba^* animals (*n* = 4, *p* < 0.0001, *t* test). Moreover, in both mutants, ∼25% of the hair cells did not exhibit any measurable current under these recording conditions.

**Table 1.** Analysis of the MET currents

	Control	*Loxhd1^T1308X/T1308X^*	*Loxhd1^Sba/Sba^*	
P7				
Number of values	6	4	4	
*I*max mean ± SD	558.3 ± 71.8 pA	484.0 ± 56.9 pA	473.3 ± 98.1 pA	
P11				
Number of values	5	8	4	
*I*max mean ± SD	395 ± 85.4 pA	24 ± 16.5 pA	25 ± 18 pA	
	*Loxhd1*^*T1308X*/+^	*Loxhd1^T1308X/T1308X^*	*Loxhd1*^*Sba*/+^	*Loxhd1^Sba/Sba^*
P9				
Number of values	14	12	6	5
*I*max mean ± SD	400 ± 94 pA	162 ± 104 pA	402 ± 104 pA	114 ± 40 pA
X mid-activation ± SD	0.756 ± 0.206 µm	0.630 ± 0.135 µm	0.809 ± 0.178 µm	0.800 ± 0.093 µm
tau 1 ± SD	0.687 ± 0.256 ms	0.779 ± 0.227 ms	0.507 ± 0.158 ms	0.530 ± 0.293 ms
tau 2 ± SD	6.96 ± 4.74 ms	12.21 ± 10.44 ms	2.77 ± 3.77 ms	1.66 ± 1.73 ms
% Adapted ± SD	85.3 ± 14.6%	85.3 ± 14.6%	88.3 ± 9.2%	88.4 ± 12.6%

The values of the maximum currents elicited by fluid jet at P7 and P11 are indicated, together with the maximum currents and kinetic parameters measured at P9 using stiff-probe stimulation of the *Loxhd1*-PLAT10 mutants. *I*max, Maximum current.

To determine whether the kinetic properties of the residual current were modified, we focused on the transition time point P9. At this age, most hair cells were expected to be affected while still maintaining a MET_max_ current amplitude compatible with kinetic studies. IHCs were mechanically stimulated with a glass probe according to a step stimulus protocol (Fig. 5*C*,*D*, Table 1). In heterozygotes of both mutant strains, a MET_max_ of 400 ± 99 pA (*n* = 21) was induced by deflection, which adapted first with a fast phase and then a slow phase. In homozygous mutant animals, the MET_max_ at P9 was reduced to 162 ± 104 pA (*n* = 12) in *Loxhd1^Sba/Sba^* animals and to 114 ± 40 pA (*n* = 5) in *Loxhd1^T1308X/T1308X^* animals (Fig. 4*D*). The normalized current/displacement relationship (n.s., *p* = 0.078, Student's unpaired *t* test), the fast and slow adaptation time constants (including the comparison of heterozygotes and homozygotes between strains; n.s., *p* = 0.069, Student's unpaired *t* test), and the extent of adaptation were not altered between genotypes (*Loxhd1*^*T1308X*/+^, *n* = 15; *Loxhd1^T1308X/T1308X^*, *n* = 5; *Loxhd1*^*Sba*/+^, *n* = 6; and *Loxhd1^T1308X/T1308X^*, *n* = 11; n.s., paired *t* test; Fig. 5*E–I*). In summary, these data provide evidence that mutations affecting LOXHD1-PLAT10 induce a hair-cell MET defect between P7 and P11 that is characterized by a drastic loss of MET_max_ without modification of the kinetic properties.

### Hair bundle morphology and TL number were preserved in *Loxhd1*-PLAT10 mutants

**Figure 6.**
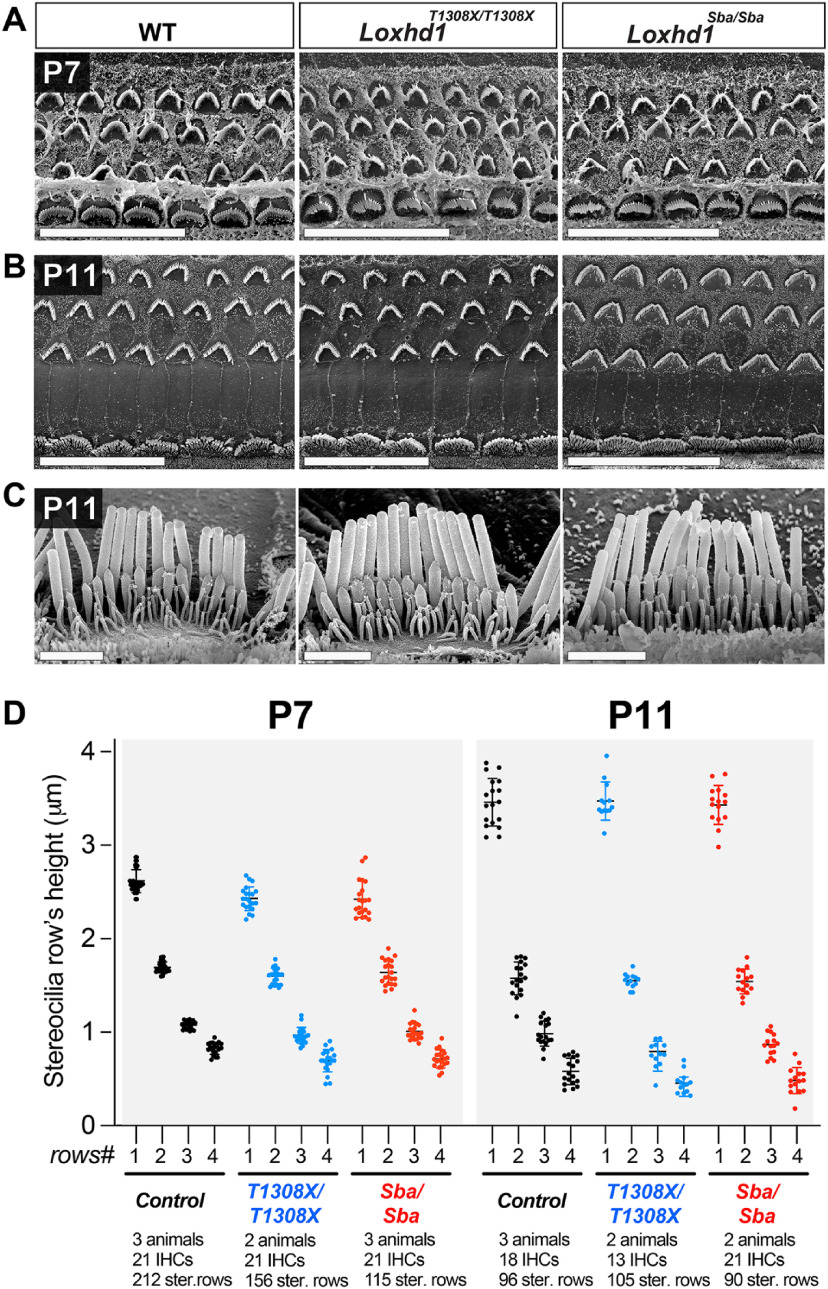
The hair bundle staircase morphology was preserved in *Loxhd1^T1308X/T1308X^* and *Loxhd1^Sba/Sba^* mutants. ***A***, ***B***, Low-magnification scanning EM micrographs of P7 (***A***) and P11 (***B***) apical–medial cochlear turns showing a normal morphology of the hair bundles in *Loxhd1^T1308X/T1308X^* and *Loxhd1^Sba/Sba^*. Scale bars, 20 µm. ***C***, High-magnification scanning EM micrographs of P11 apical IHC hair bundles in *Loxhd1^T1308X/T1308X^* and *Loxhd1^Sba/Sba^* specimens. Scale bars, 2 µm. ***D***, Stereociliary row heights from individual IHCs at P7 and P11 determined from scanning EM pictures. The mean heights and SD for each row are displayed.

**Figure 7.**
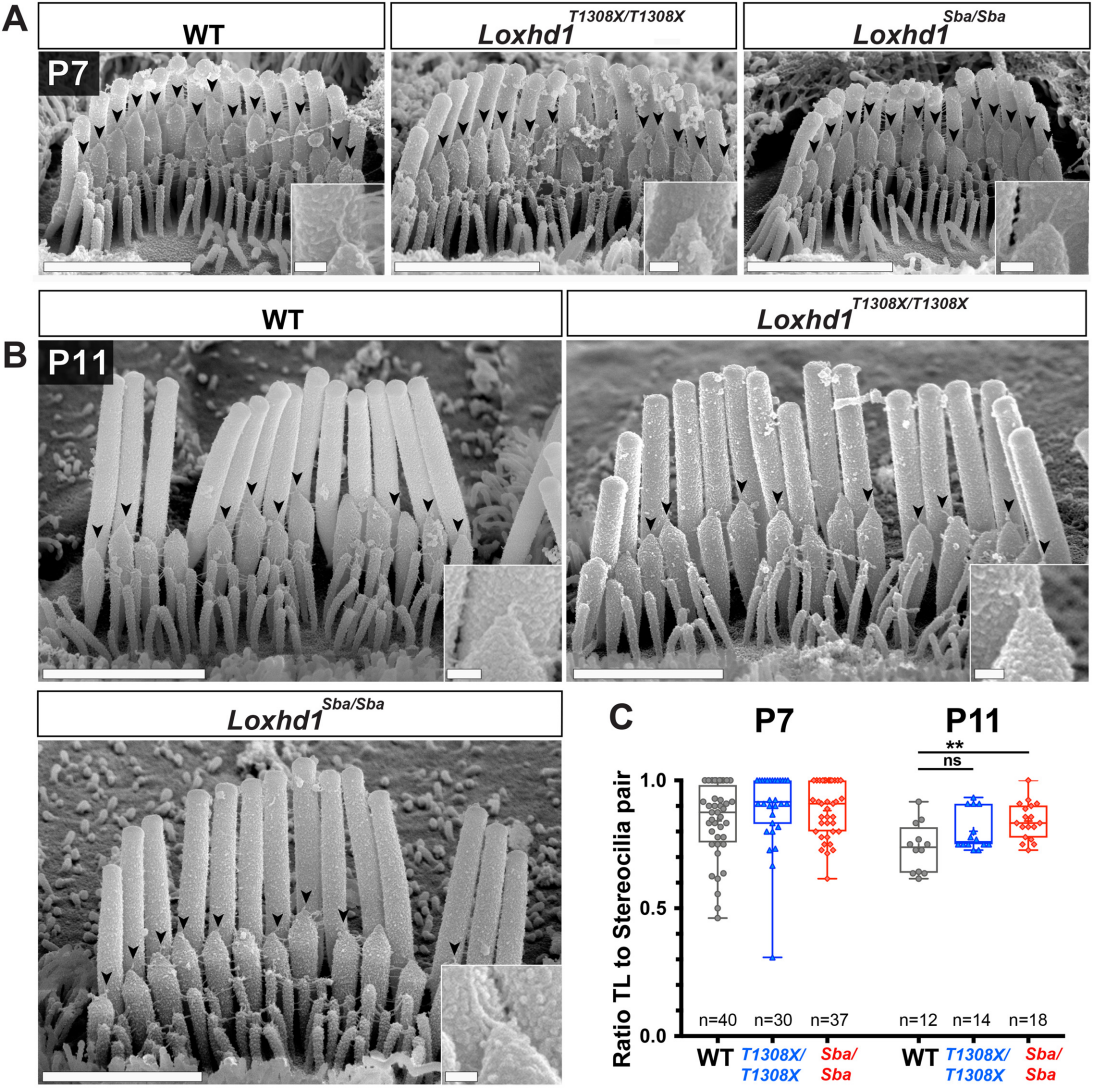
The number of tip links at P7 and P11 was not reduced in the *Loxhd1^T1308X/T1308X^* and *Loxhd1^Sba/Sba^* mutants. ***A***, ***B***, Tip links joining the first (tallest) and second stereociliary rows were visible (arrowheads and inset) on high-magnification scanning EM micrographs of P7 (***A***) and P11 (***B***) apical–medial IHCs. Scale bars: main pictures, 2 µm; insets, 100 nm. ***C***, The ratio of TL to stereociliary pairs was not reduced in *Loxhd1* mutants compared with WT littermates at P7 and P11. The plot shows the average ratios of “*n*” IHCs in a 1−99% whisker plot.

The loss of the MET current detected between P7 and P11 in *Loxhd1* mutants could potentially be explained by a progressive hair bundle structural defect that might prevent MET channel stimulation. To explore this possibility, we performed scanning EM on apical cochlear turns at P7 and P11. At both time points, hair bundles from *Loxhd1^T1308X/T1308X^* and *Loxhd1^Sba/Sba^* were indistinguishable from those of control animals at both low magnification (Fig. 6*A*,*B*) and high magnification (Fig. 6*C*). The staircase organization was also preserved, and the height of stereocilia rows was comparable across genotypes, with a minor reduction observed in rows 3 and 4 at P7 and P11 (Fig. 6*D*) in *Loxhd1^T1308X/T1308X^* animals [P7, row 3: −11.7%, Mann−Whitney test (*p* < 0.0001); P7, row 4: −17.2% (*p* < 0.0001); P11, row 3: −19.3% (*p* = 0.0017); and P11, row 4: −18.4% (*p* = 0.0138)] and *Loxhd1^Sba^*^/^*^Sba^* animals (P7, row 3: −9.0% (*p* = 0.0014); P7, row 4: −16.0%; P11 row 3: −9.5% (*p* = 0.0118)]. In addition, the number of TLs bridging rows 1 and 2 was not reduced in *Loxhd1^T1308X/T1308X^* or *Loxhd1^Sba/Sba^* animals compared with WT animals at P7 and P11 (Fig. 7*A–C*), with even more TLs detected at P11 in *Loxhd1^Sba/Sba^* versus control specimens (9.47% increase; *p* < 0.005, two-tailed Mann−Whitney test). Finally, we did not observe any loss of the prolate shape of the second-row stereocilia tips, supporting the conclusion that TLs are present in these structures (Tilney et al., 1988). We conclude that the MET phenotype is not caused by a gross structural defect in the mechanosensory organelle, as assessed using scanning EM.

### LHFPL5 and Harmonin are maintained in *Loxhd1*-PLAT10 mutant hair bundles

**Figure 8.**
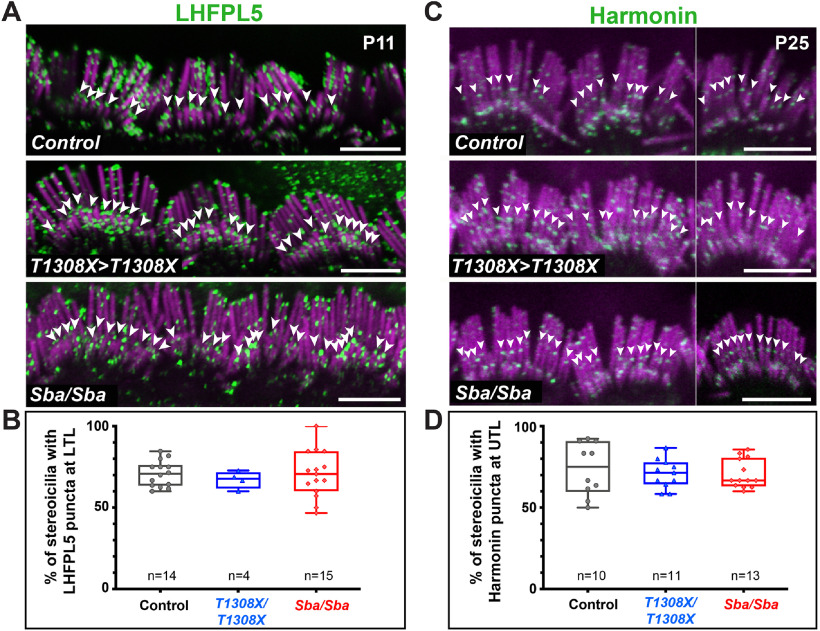
LHFPL5 and Harmonin were maintained in *Loxhd1*-PLAT10 mutant hair bundles. ***A***, Immunofluorescence staining of P11 IHC hair bundles with an anti-LHFPL5 antibody (green) present at the LTL area (arrowheads) and F-actin (magenta). Scale bars, 5 µm. ***B***, Percentage of stereocilia with LFHPL5 puncta at the LTL insertion point at the middle stereocilia row. ***C***, Immunofluorescence staining of P25 IHC hair bundles (assembly of different examples) with anti-Harmonin (green) detected at the UTL area (arrowheads) and F-actin (magenta). Scale bars, 5 µm. ***D***, Percentage of stereocilia with Harmonin puncta at the UTL insertion point at the tall stereocilia row.

We then studied the hair bundle at the molecular level to determine whether the protein complexes at each TL insertion point were maintained in mutant IHCs at P11 or later. First, we investigated the LTL insertion point, where the MET channel complex localizes. Using a validated antibody, we investigated the localization of LHFPL5, an essential component of the MET channel complex located at the tip of transducing stereocilia (Li et al., 2019). LHFPL5 exhibited a similar localization across genotypes and was identified at the LTL in each case (Fig. 8*A*), as reported previously (Xiong et al., 2012; Li et al., 2019). We determined the percentage of stereocilia with LFHPL5 puncta at the LTL insertion point, focusing on the second row. No statistical differences were found between genotypes (Fig. 8*B*; n.s., two-tailed Mann−Whitney test).

Second, we evaluated the presence of the UTL complex, which anchors the TL to the cytoskeleton. We focused our analysis on the *Ush1c* gene product, Harmonin, which is the most abundant protein of this complex and for which a validated antibody exists (Grillet et al., 2009a; Grati and Kachar, 2011). We found that even at a late age (P25), the Harmonin signal was clustered at the UTL insertion point in the tallest stereociliary row of IHCs in the two *Loxhd1* mutants (Fig. 8*C*). Within a given hair bundle, the percentage of stereocilia with Harmonin puncta at the UTL insertion point was similar between genotypes (n.s., two-tailed Mann−Whitney test; Figs. 8*D*). Inactivation of the *lhfpl5* and *Ush1c* genes led to early mechanotransduction defects in cochlear hair cells, which were detectable as early as P1–P3 (Michalski et al., 2009; Xiong et al., 2012). Other than LOXHD1, the only protein with a late-onset hair-cell mechanotransduction phenotype is the MET channel complex component TMC1 (Kim and Fettiplace, 2013; Asai et al., 2018). We attempted to determine whether TMC1 was maintained in *Loxhd1*-PLAT10 mutants by immunolocalization at P11. We found no difference between the homozygous mutants and controls; however, the staining was not confined to the stereociliary tips and, unlike in previous work, some amount of staining remained in the *Tmc1^KO^* animals; thus, the TMC1 localization was unconvincing (data not shown; Mahendrasingam and Furness, 2019). In summary, we conclude that several TL complex proteins are maintained in the hair bundle when LOXHD1-PLAT10 is mutated, suggesting that the TL complexes are not disassembled at the time of the MET defect.

**Figure 9.**
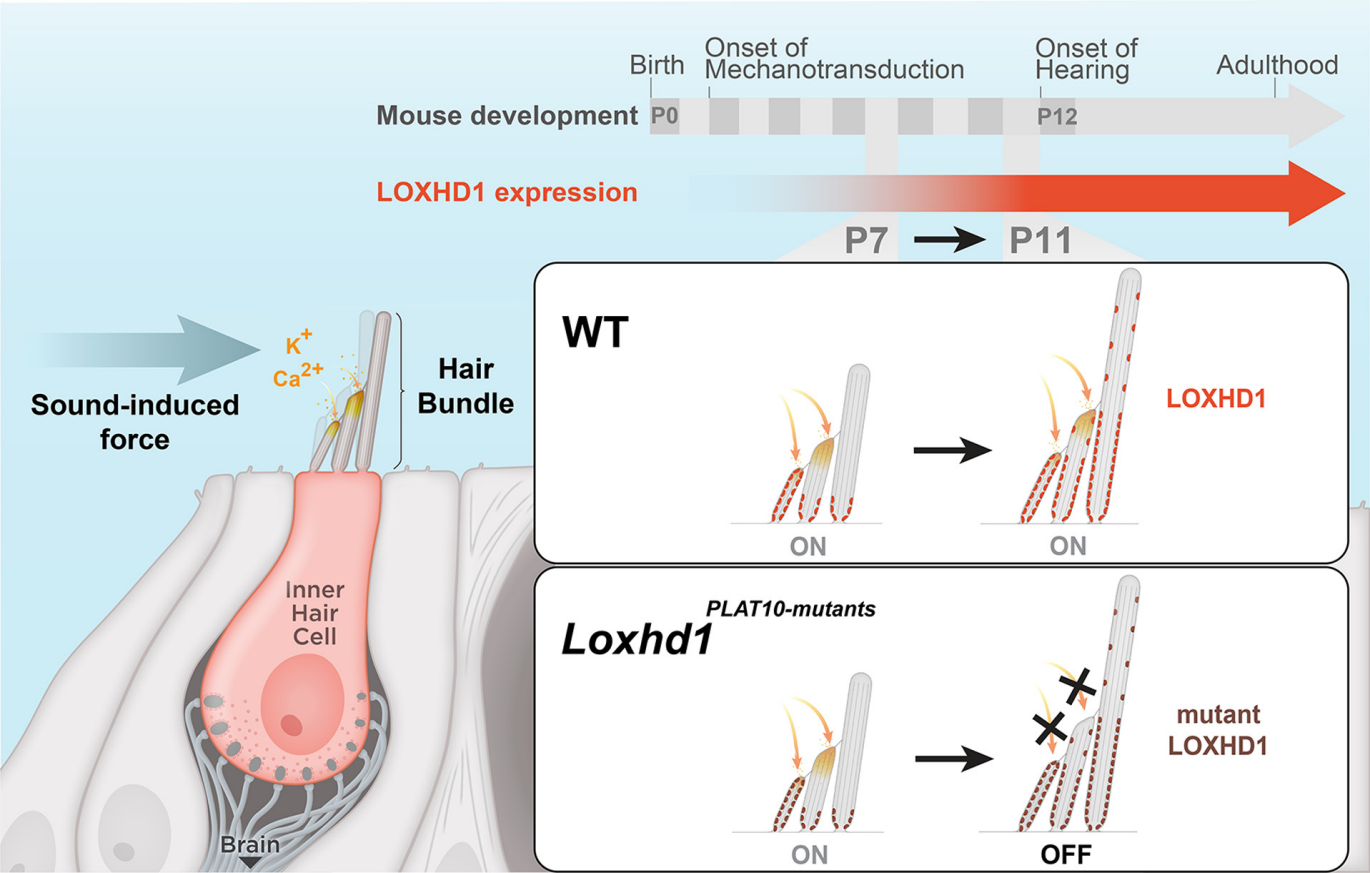
Graphical summary. Mechanotransduction of IHCs is affected in mice carrying mutations in the *Loxhd1* gene specifically, in one of the exons encoding the PLAT10 repeat. The mechanotransduction defect manifests only after P7 and leads to hearing loss. The MET defect does not affect the hair bundle morphology or the maintenance of Harmonin and LHFPL5 in the bundle, suggesting that MET channels are present but not activatable.

## Discussion

Mutations in LOXHD1 lead to recessive hearing loss in humans (DFNB77) and mice; however, its function remains unknown. We showed that LOXHD1 is required for mechanotransduction in mouse IHCs. Interestingly, this dependence began after the first postnatal week. In fact, mutations affecting LOXHD1 at the PLAT10 repeat led to a 95% reduction in the MET current between P7 and P11 (Fig. 9). The late developmental onset of the MET phenotype was correlated with the temporal progression of postnatal LOXHD1 expression/localization in the hair bundle. LOXHD1 localizes along the stereocilia height, with enrichment observed toward the base. The *Loxhd1* MET defect was novel, as it occurred without an altered hair bundle structure or mislocalization of key upper (Harmonin) or lower (LHFPL5) TL complex proteins. Together, these results suggest that the mechanotransduction machinery is present in the hair bundle of *Loxhd1* mutants but does not function properly. Thus, LOXHD1 highlights the importance of the stereocilia microenvironment for mechanotransduction function. Furthermore, our findings reveal a time window during which late developmental changes in mechanotransduction occur at the molecular level.

### Variability of the *Loxhd1/DFNB77* auditory phenotypes

We used two mouse models carrying mutations in the same exon to robustly study *Loxhd1* function in hair cells. This approach also allowed us to compare the outcomes of the missense mutation in *Loxhd1^Sba^* (I1342N) with those of the nonsense mutation followed by a short deletion in *Loxhd1 ^T1308X^* (p.T1308X_ P1420del). Unexpectedly, hearing loss was attenuated in *Loxhd1^T1308X/T1308X^* compared with *Loxhd1^Sba/Sba^* mice, with residual CM at P24, and residual ABR and DPOAE responses remaining stable between P21 and P60 in some animals. The reason for this low penetrance is unclear and could have different origins. First, the genetic background of the strain could influence the *Loxhd1* phenotype. The *Loxhd1^T1308X^* allele was produced on a mixed background (OLA129, SLJ, C57BL/6) and was subsequently backcrossed for 14 generations onto C57BL/6J, which should result in a 99.99% C57BL/6J backcrossed strain (Flurkey et al., 2009). However, we cannot exclude the possibility that a *de novo* mutation or a gene modifier influencing the phenotypic expression might be present in the vicinity of the *Loxhd1* gene in some animals. Second, the *Loxhd1^T1308X/T1308X^* strain may be more resistant to hearing loss because of the nature of its mutation. In fact, we discovered an mRNA surveillance mechanism occurring exclusively in the *Loxhd1^T1308X/T1308X^* hair cells. The NAS surveillance mechanism promotes splicing around the mutated exons and maintains open reading frames (Valentine, 1998; Liu et al., 2001; Wang et al., 2002). NAS has previously been implicated in an attenuated phenotype induced by a nonsense mutation in a gene required for vision (Barny et al., 2018), but never in genes required for hearing. Here, we showed that hair cells are capable of using NAS. Because of the repeated structure of LOXHD1, NAS may regain some level of functioning, thereby attenuating hearing loss.

In humans, LOXHD1/DFNB77 mutations lead to hearing loss with a wide range of onset and severity—a phenomenon that remains unexplained (Grillet et al., 2009b; Edvardson et al., 2011; Mori et al., 2015; Minami et al., 2016; Wesdorp et al., 2018; Maekawa et al., 2019). Interestingly, the nature of the pathogenic mutations in *LOXHD1*/DFNB77 is largely biased toward nonsense mutations (Azaiez et al., 2018). NAS could be the underlying reason for the variable onset and progression of hearing loss in patients with DFNB77 (Mori et al., 2015; Wesdorp et al., 2018; Maekawa et al., 2019) and could also potentially be used by other deafness genes with repeated domains to bypass nonsense mutations.

### LOXHD1 expression in hair cells

To study LOXHD1 expression in the hair bundle, we used an *in vitro*-validated antibody against PLAT11/12 with the intention to also validate it *in vivo* using the *Loxhd1^T1308X^* allele. However, the anti-PLAT11/12 antibody retained staining in the *Loxhd1^T1308X/T1308X^* hair bundle, likely because of the occurrence of NAS mRNA splicing. Therefore, the anti-PLAT11/12 antibody results must be considered with caution because LOXHD1 specificity still requires *in vivo* confirmation. Ultimately, a mouse model in which the entire *Loxhd1* gene (160 kb, 41 exons) is inactivated will be required to prevent compensation by splicing or the usage of alternative translational start sites and to prevent putative cross-reactivity between the PLAT repeats. Nevertheless, an indication that the anti-PLAT11/12 antibody exhibits a specific LOXHD1 signal arises from the gradual increase in the hair bundle staining observed between P7 and P11, which correlates with the occurrence of the MET phenotype. This signal is distributed along the height of the stereocilia, with enrichment at the lower stereocilia portions of rows 1 and 2, forming a ring around the actin core. Since LOXHD1 is expressed in all stereocilia rows, it could affect mechanotransduction from either side of the TL insertion points.

### LOXHD1 and mechanotransduction

As suggested by CM measurements, the MET function of hair cells is defective in *Loxhd1^Sba/Sba^* and *Loxhd1^T1308X/T1308X^* mice. Using *ex vivo* whole-cell voltage clamping of apical IHCs (whose location corresponded to a 6–10 kHz frequency range in the adult mouse), we timed the MET phenotype onset between P7 and P11 in the two mutants. At P11, a maximum MET current of 25 pA could be elicited by mechanical stimulation. This receptor current would correspond to a maximum depolarization of ∼2.5 mV (considering a hair-cell membrane resistance of 100 MΩ), which is unlikely to have a major effect on the calcium current that drives synaptic release. The loss of MET current will reduce the resting current, leading the IHC to hyperpolarize by ∼10 mV, and further lower the effect of the 2.5 mV depolarization (Johnson et al., 2011). However, we have recorded ABRs between 6 and 12 kHz in *Loxhd1^Sba/Sba^* animals at P21, providing strong evidence that a population of IHCs conserved some MET activity *in vivo*. It is possible that our mutant hair cells are particularly sensitive to mechanical damage and could be irreversibly affected during *ex vivo* sample preparation. Alternatively, the *ex vivo* mechanical stimulation applied to the hair bundle may not fully reproduce the *in vivo* stimulation. Nevertheless, mutations in the LOXHD1 PLAT10 repeat reduce the IHC current amplitude without modifying kinetic properties. This could result from either a reduction in the total number of MET channels or a reduction in the number of activable MET channels. Therefore, we investigated the localization of MET machinery components in the mutant. Using scanning EM, we found that TLs still connected stereocilia rows at P11. Furthermore, both the UTL complex protein Harmonin and the LTL complex protein LHFPL5 were correctly positioned in mutant IHCs. However, other components of the TL complexes that were not evaluated here, such as TMIE, TMC1, CIB2, MYO7A, or USH1G, could be affected. Further testing will be necessary to ensure that all components of the MET complex are present.

Alternatively, LOXHD1 may not be responsible for targeting or maintaining these components; rather, it may underlie their activity in mature hair bundles. LOXHD1 may function by directly interacting with MET complex proteins or by indirectly regulating the mechanical properties of the stereocilia membrane. We will investigate these possibilities in future work.

Finally, regardless of the molecular role of LOXHD1, it is remarkable that the MET phenotype occurred only after P7. As observed using our anti-PLAT11/12 antibodies, LOXHD1 was already detectable in the hair bundle by P7. This disconnect may reflect a transition step in which interactions are being established that require a certain level of the LOXHD1 protein. It is also possible that an alternative protein performs the LOXHD1-PLAT10 function before P7, with likely candidates including LOXHD1 protein isoforms that do not contain PLAT10. We will explore this hypothesis in the future and test whether a null allele has a more severe phenotype.

In this study, we primarily focused on the IHCs, but LOXHD1 is expected to be similarly required for MET in OHCs, which express LOXHD1 (mRNA and protein) and are functionally affected by the *Loxhd1*-PLAT10 mutations (CM and DPOAE).

In conclusion, we have added LOXHD1 to the short list of proteins required for hair-cell MET. Mutations in PLAT10 led to an IHC MET defect that occurred only after the first postnatal week. Furthermore, this defect did not affect the hair bundle structure or the maintenance of Harmonin and LHFPL5 in the bundle, suggesting that MET channels are present but not activatable. Understanding this mode of MET regulation is critical, as it underlies both congenital and age-related forms of hearing loss in humans.
